# Development of Minicircle Vectors Encoding COL7A1 Gene with Human Promoters for Non-Viral Gene Therapy for Recessive Dystrophic Epidermolysis Bullosa

**DOI:** 10.3390/ijms222312774

**Published:** 2021-11-26

**Authors:** Xianqing Wang, Fatma Alshehri, Darío Manzanares, Yinghao Li, Zhonglei He, Bei Qiu, Ming Zeng, Sigen A, Irene Lara-Sáez, Wenxin Wang

**Affiliations:** 1 Charles Institute of Dermatology, School of Medicine, University College Dublin, D04 V1W8 Dublin, Ireland; xianqing.wang@ucdconnect.ie (X.W.); dario.sandoval@ucd.ie (D.M.); yinghao.li@ucdconnect.ie (Y.L.); zhonglei.he@ucd.ie (Z.H.); bei.qiu@ucdconnect.ie (B.Q.); zengming003@163.com (M.Z.); sigen.a@ucd.ie (S.A.); 2 College of Science, Princess Nourah Bint Abdulrahman University, Riyadh 11671, Saudi Arabia; faoalshehri@pnu.edu.sa; 3 Department of Dermatology, The First Affiliated Hospital of Jinan University, Guangzhou 510630, China

**Keywords:** non-viral gene therapy, recessive dystrophic epidermolysis bullosa (RDEB), COL7A1 gene, minicircle DNA, CMV, EF1α, COL7A1 tissue specific promoter

## Abstract

Recessive dystrophic epidermolysis bullosa (RDEB) is a rare autosomal inherited skin disorder caused by mutations in the COL7A1 gene that encodes type VII collagen (C7). The development of an efficient gene replacement strategy for RDEB is mainly hindered by the lack of vectors able to encapsulate and transfect the large cDNA size of this gene. To address this problem, our group has opted to use polymeric-based non-viral delivery systems and minicircle DNA. With this approach, safety is improved by avoiding the usage of viruses, the absence of bacterial backbone, and the replacement of the control viral cytomegalovirus (CMV) promoter of the gene with human promoters. All the promoters showed impressive C7 expression in RDEB skin cells, with eukaryotic translation elongation factor 1 α (EF1α) promoter producing higher C7 expression levels than CMV following minicircle induction, and COL7A1 tissue-specific promoter (C7P) generating C7 levels similar to normal human epidermal keratinocytes. The improved system developed here has a high potential for use as a non-viral topical treatment to restore C7 in RDEB patients efficiently and safely, and to be adapted to other genetic conditions.

## 1. Introduction

Epidermolysis Bullosa (EB) is a family of genetic skin fragility disorders associated with the lack of structural integrity in the skin. The most severe form of EB is recessive dystrophic epidermolysis bullosa (RDEB), attributed to mutations in the COL7A1 gene, that encodes the skin structural protein type VII collagen (C7) [1]. Consequently, the compromised skin integrity in RDEB leads to characteristic clinical manifestations of severe and chronic blistering, mitten deformities of the hands and feet, and mucosal and organ lesions with an increased risk of developing an aggressive form of squamous cell carcinoma [2,3]. Currently, there is no effective treatment available for RDEB, and interventions are focused on managing symptoms in order to improve patients’ quality of life. Since RDEB is a monogenic disease and C7 is a protein with a long half-life, which ranges from one to two months, gene replacement is a good therapeutic option for RDEB [4]. However, the large cDNA size of the COL7A1 gene (8.9 kb) has a negative impact on gene delivery, which challenges the non-viral and viral vectors’ packaging capacity, thus limiting the transfection efficiency [5,6,7]. There are more than 30 clinical trials ongoing of gene therapies for RDEB, but only one (B-VEC), a phase III clinical trial conducted by Krystal Biotech, directly delivers the functional version of COL7A1 gene into the skin cells to restore it. In this case, the encapsulation and delivery were possible by employing a modified herpes simplex virus (HSV) as the gene vector.

The success of gene therapy treatments mostly depend on the development of a vector that can selectively and efficiently deliver the COL7A1 gene to target cells with high levels of gene expression, long term correction, lack of immune-mediated response, and minimal toxicity [8,9,10,11]. Compared to viral vectors, non-viral systems offer the advantages of minimal immunogenicity, non-tumorigenicity, cost-effective manufacturing, and high loading capacity [12,13,14]. Among them, cationic polymers and cationic lipids are the main representatives. Although their theoretical DNA payload capacity is relatively high, cationic lipids still have limitations, established by Campeau et al. as 12 kb [15]. Moreover, Janich et al. achieved high transfection efficiency using a modified cationic lipidic vector with a 15.5 kb plasmid DNA in HEK293T [16]. In the past decade, our group has been developing cationic polymers able to encapsulate and deliver large plasmids (15.8–16 kb) into different cell lines, including RDEB keratinocytes (RDEBK), with high transfection efficiency and low toxicity [17,18]. Recently, an outstanding member of a new family of those polymers was developed by our group and was commercialized under the name of BrBPERfect.

Along with successful delivery of the DNA to lead to good target protein expression, RDEB offers an additional challenge. As this disease requires long-term treatment, repeated applications of a therapeutic agent could potentially cause immunogenicity and irritation problems. With this in mind, a treatment that could generate long-term C7 expression is highly desirable, in order to reduce the frequency of applications in the severely fragile skin.

Minicircle DNA (MC) technology involves small circular DNA plasmids where the antibiotic selection marker and the prokaryotic origin of the replication sequences have been removed. The use of MC increases the therapeutic safety profile, as it can reduce any potential enterobacteria horizontal gene transfer [19] and the removal of the unmethylated CpG motifs in the origin of replication leads to higher levels of transgene products by reducing the activation of an immune response and silencing by methylation of DNA [20,21]. Thus, the development of a minicircle system would provide several advantages to overcome the previously mentioned limitations by expanding the DNA loading capacity and increasing then the transfection efficiency complexed to the vector, leading to more sustained expression and improved therapeutic safety profile compared to traditional plasmids. Our previous study, using a minicircle DNA (MCC7) encoding the COL7A1 gene under the control of the viral cytomegalovirus (CMV) promoter, showed that in vitro C7 expression lasts longer compared to the conventional plasmid (16 kb) [18]. Furthermore, an in vivo study using MCC7 showed significant levels of C7 restoration in a RDEB skin graft mouse model detected after 30 days of transfection [9].

Although viral promoters usually contribute to high gene expression, the usage of them carries the risk of transgene silencing and DNA methylation. In fact, in 2010, this problem led to a gene therapy clinical trial for X-linked chronic granulomatous disease (X-CGD) ending with a negative impact [22]. Moreover, when viral promoters are used to control transgene expression, systemically administered plasmid vectors can induce both humoral and cell-mediated immune responses [23,24,25]. Currently, there are many studies focusing on human promoter optimization, which make the system more resistant to silencing and, therefore, increase its safe use as a gene therapy tool in the clinic [26,27,28,29].

In this study, we selected the human COL7A1 tissue specific promoter (C7P) and the ubiquitous human eukaryotic translation elongation factor 1 α (EF1α) promoter, both commonly used in skin therapy, to replace the CMV promoter in MCC7, and then formulate the minicircle DNA with a highly efficient non-viral vector into nanoparticles for transfection [28,30]. Our hypothesis is that transfection of MCC7 with human promoters mediated by the non-viral DNA transfection reagent BrPERfect could lead to an efficient and much safer gene replacement treatment for RDEB patients, which is more promising to be translated from ‘bench to bedside’.

## 2. Results

### 2.1. MN511C7 Parental Plasmid Generation

**Figure 1:**
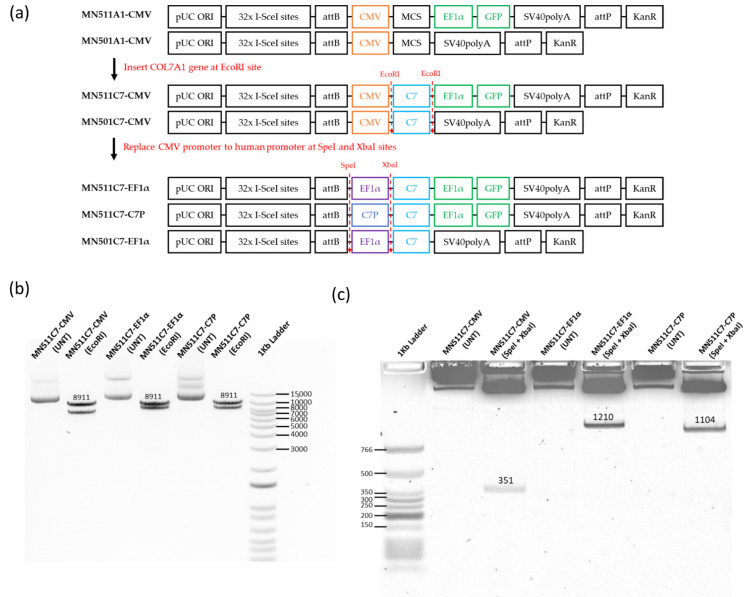
MN511C7 parental plasmid construct confirmation. (**a**) Scheme of type VII collagen (C7) minicircle parental plasmid construction. (**b**) MN511C7- control viral cytomegalovirus (CMV) plasmid (MN511C7-CMV), MN511C7- eukaryotic translation elongation factor 1 α (EF1α) plasmid (MN511C7- EF1α), and MN511C7-COL7A1 tissue specific promoter (C7P) plasmid (MN511C7-C7P) were digested by EcoRI restriction enzyme digestion, and the digested products were separated on a 0.8% agarose gel. All the plasmids had an 8911 kb C7 band. (**c**) MN511C7-CMV, MN511C7-EF1α, and MN511C7-C7P were digested by SpeI and XbaI dual restriction enzyme digestion. CMV (351 bp), EF1α (1210 bp) and C7P (1104 bp) bands on a 0.8% agarose gel confirmed the successful promoter replacements.

In order to develop a gene replacement therapy for RDEB, two human promoters were selected to compare with the CMV promoter. The human COL7A1 promoter (C7P) sequence and EF1α sequence were present in the already synthesized pEX-K-C7P and pEX-K-EF1α plasmids. SpeI and XbaI restriction enzyme sites were added to both promoters at the 5′ and 3′ ends respectively for further construction. The full human COL7A1 gene sequence cut from pcDNA3.1C7 plasmid was firstly inserted into MN511A1-CMV to obtain the MN511C7-CMV plasmid. Then, using SpeI and XbaI dual restriction enzyme digestion, the CMV promoter was removed from the MN511C7-CMV plasmid, and the human promoters cut from pEX-K vectors were inserted to generate MN511C7-C7P and MN511C7-EF1α (Figure 1a and Figure S1a). COL7A1 sequence and human promoter sequence insertion were confirmed by restriction enzyme digestion and sequencing analysis. MN511C7-C7P contained the human COL7A1 tissue specific promoter, attB and attP sites, the human COL7A1 gene, and GFP genes, with its total size being 15,414 bp. MN511C7-EF1α was 16,819 bp, containing human EF1α promoter, attB and attP sites, the human COL7A1 gene and GFP genes. The COL7A1 sequence of each plasmid was confirmed by EcoRI restriction enzyme digestion, which cut MN511C7-C7P into an 8911 bp band (COL7A1) and a 6503 bp residual band, and cut MN511C7-EF1α into an 8911 bp band (COL7A1) and a 7908 bp residual band (Figure 1a,b). The XbaI and SpeI dual restriction enzyme digestion was used to confirm the size of each promoter: the 351 bp band on agarose gel corresponded to the CMV promoter, the 1210 bp band to the EF1α promoter, and the C7P promoter to the 1104 band (Figure 1a,c).

### 2.2. In Vitro Transfection Efficiency and Cell Viability on RDEB Cells Using Commercial Transfection Reagents

**Figure 2:**
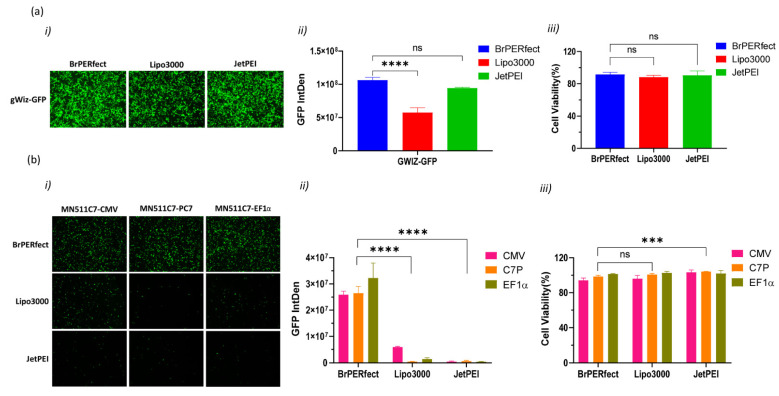
DNA transfection reagent selection for transfection of recessive dystrophic epidermolysis bullosa keratinocytes (RDEBK). (**a**) Transfection of gWiz-GFP plasmid by using BrPERfect (blue), Lipofectamine 3000 (red) and jetPEI (green) reagents 48 h post-transfection. (**a**(i)) Fluorescence microscopy images of GFP expression captured with a 4X objective. (**a**(ii)) Integrated fluorescence intensity related to GFP expression semi-quantified using ImageJ software. (**a**(iii)) Cell viability of RDEBK cells tested using alamarBlue™ assay. (**b**) Transfection of MN511C7-CMV (blue), MN511C7-C7P (red) and MN511C7-EF1α (green) plasmids by using BrPERfect transfection reagent. (**b**(i)) Fluorescence microscopy images of GFP expression captured with 4X objective (**b**(ii)) Integrated fluorescence intensity related to GFP expression from MN511C7 plasmids, semi-quantified using ImageJ software. (**b**(iii)) Cell viability of transfected RDEBK cells with MN511C7 plasmids was tested using alamarBlue™. All data was collected from 3 replicates of 3 independent experiments and presented as means ± SD (*n* = 3). *** *p* < 0.001; **** *p* < 0.0001; ns: not significant, as compared to BrPERfect. Fluorescence microscopy images are representative of three independent experiments (*n* = 3). IntDen: integrated density; ns: not significant.

The newly generated plasmids were quite large (MN511C7-CMV: 15,974 bp, MN511C7-C7P: 15,414 bp, and MN511C7-EF1α: 16,819 bp). To deliver such large C7 plasmids (around 16 kb) efficiently, we selected and compared three non-viral commercial reagents for transfection on RDEB cells, including BrPERfect, Lipofectamine 3000 and jetPEI. All of them were formulated with gWiz-GFP plasmids or each MN511C7 plasmid, following their user manuals. Fluorescence images were taken 48 h post-transfection and processed for semi-quantitative analysis using Image J. The GFP expression from gWiz-GFP plasmid showed that all commercial reagents had very high transfection efficiency and good cytocompatibility on RDEBK (Figure 2a(i–iii)). BrPERfect and jetPEI, two polymer-based DNA vectors, showed no statistical differences for transfection efficiency on RDEBK cells (*p* = 0.0506), and their transfection ability was better than Lipofectamine 3000 (*p* ˂ 0.0001 when compared with BrBPERfect and *p* = 0.0002 when compared with jetPEI), which is a liposome (Figure 2a(i,ii)). However, all three reagents showed limited transfection efficiency on the RDEB fibroblasts (RDEBF) cells. Compared to GFP-positive RDEBK cells, only few RDEBF cells expressed GFP after transfection (Figure S2a). As mentioned earlier, the large size of the C7 sequence is the main obstacle for gene replacement therapy of RDEB. When we transfected RDEBK cells with MN511C7 plasmids, the transfection efficiency of all the reagents was reduced. Thus, less GFP positive cells were found when transfecting them with MN511C7 plasmids than with the gWiz-GFP plasmid. Particularly, the number of GFP-positive cells transfected with Lipofectamine 3000 and jetPEI sharply dropped. However, BrPERfect still showed high levels of transfection efficiency on RDEBK cells, which indicated its outstanding performance on DNA delivery (Figure 2b(i,ii)). All the transfections conducted here showed over 80% of cell viability in relation to the untreated cells (Figure 2a,b(iii) and Figure S2b). Based on these results, BrPERfect was the transfection reagent selected for further assays.

### 2.3. Comparison of Type VII Collagen Expression Level under CMV, C7P and EF1α Promoters

**Figure 3:**
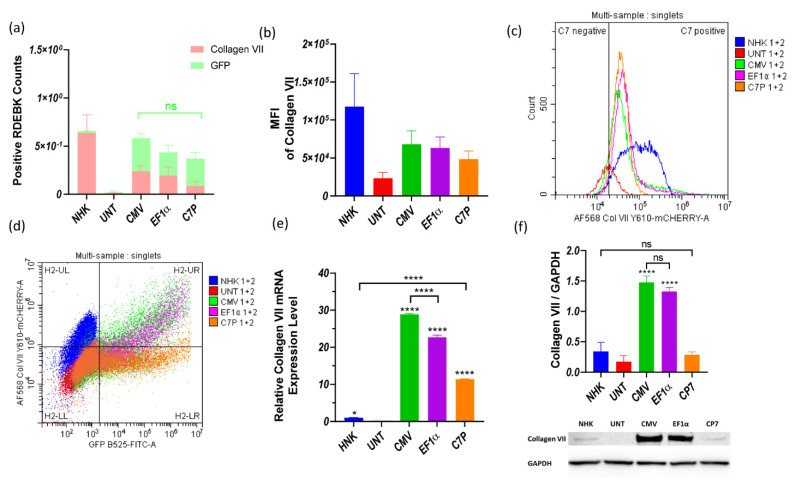
Collagen VII expression from untreated NHK and RDEBK cells transfected with plasmid/BrPERfect complexes. (**a**) NHK and RDEBK cells positive for GFP and C7 expression after transfection with MN511C7-CMV, MN511C7-EF1α and MN511C7-C7P plasmids. (**b**) Mean fluorescence intensity of C7 of NHK and treated RDEBK cells. (**c**) Distribution of positive NHK cells and RDEBK cells for C7 expression. (**d**) Gate strategy of the flow cytometry results and the dot plot of single cells. (**e**) COL7A1 gene transcription level of RDEBK and NHK cells was compared by RT-qPCR. (**f**) C7 protein monomer (290 KDa) was detected by western blot, and its expression level was semi-quantitated and normalized with the loading control protein GAPDH. UNT: untreated RDEBK cells; CMV: MN511C7-CMV transfected RDEBK cells; EF1α: MN511C7- EF1α transfected RDEBK cells; C7P: MN511C7- C7P transfected RDEBK cells; MFI: mean fluorescence intensity. For (**a**–**d**) data were collected from 3 independent experiments except for controls that data were collected from 2 independent experiments. For (**e**) data were collected from 3 replicates and (**d**) data were collected from 3 replicates of 3 independent experiments and presented as means ± SD (*n* = 3). **** *p* < 0.0001, * *p* < 0.05, as compared between different columns. ns: not significant.

Minicircle parental plasmids (MN511C7-CMV, MN511C7-EF1α, and MN511C7-C7P) were used to compare the promoters controlling C7 expression in RDEBK cells. The control plasmid consists of a traditional DNA vector whose COL7A1 transcription is regulated by a CMV promoter. Flow cytometry was firstly employed to compare the transfection efficiency induced by each plasmid. Since GFP was controlled by the EF1α promoter in all the plasmids, its expression should be the same in all cases if transfection efficiency is consistent among treatments, and our observations confirmed similar expression across the plasmids (Figure 3a). At the same time, RDEBK cells were stained with anti-C7 antibody and AlexaFluor™ 568-labeled antibody to detect C7 expression, using also untreated NHK cells as non-pathogenic control. As can also be observed in Figure 3a, the proportion of cells positive for C7 expression induced by the plasmid containing the CMV promoter (MN511C7-CMV) and induced by the EF1α promoter (MN511C7-EF1α) were one third of the NHK C7 positive cells (expressing C7 naturally) and were four times more than the cells positive for C7 expression induced by MN511C7-C7P plasmid. Moreover, the stained C7 mean fluorescence intensity (MFI) of transfected RDEBK reached about 50% of the total C7 MFI of the NHK cells in all cases, which was much higher than in the case of untreated RDEBK cells (Figure 3b). These results can be further confirmed by the distribution of cells positive for C7 and the dot plot information provided, where the total C7 expression is considerably higher and more homogenous for NHK cells (Figure 3c,d).

COL7A1 transcription and expression levels under the three different promoters were further compared by RT-qPCR and western blot. Total RNA from RDEBK cells was harvested 48 h after transfection and RT-qPCR was run, employing a comparative cycle threshold method to compare the COL7A1 transcription levels. Using the transcription level of NHK as calibrator, it was found that the RDEBK cells transfected with MN511C7-CMV produced about 30 times higher mRNA levels and MN511-EF1α about 25 times more. The mRNA level obtained from transfections with the plasmid containing C7P was the lowest among the three promoters, which was still 10 times higher than NHK cells (Figure 3e). Despite the higher COL7A1 transcription level from RDEBK cells transfected with MN511C7-C7P compared to NHK, the two cell types showed no statistical difference in C7 protein expression. Similarly, mRNA levels of C7 are significantly higher for the plasmid containing a CMV promoter compared to the plasmid with EF1α, but this is not translated to a significantly higher level of protein expression. Nevertheless, C7 protein expression under control of CMV and EF1α promoters was consistent with transcription levels, which was around five-fold of the C7 protein expression in NHK cells (Figure 3f).

### 2.4. MN501C7 Parental Plasmid Construction and Minicircle Induction

**Figure 4:**
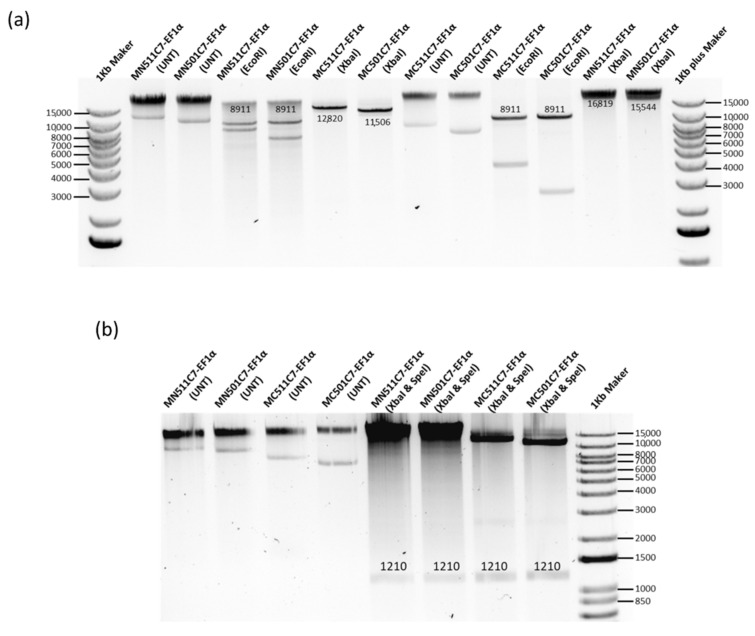
MN501C7-EF1α parental plasmid construct and MC501C7-EF1α confirmation. (**a**) MN511C7-EF1α, MN501C7-EF1α, MC511C7-EF1α, and MC501C7-EF1α were digested by EcoRI restriction enzyme to confirm the C7 sequence (8911 kb) insertion. After XbaI restriction enzyme digestion, the size of linear MC511-EF1α, and MC501C7-EF1α were about 4 kb smaller than their parental plasmids. There were no parental plasmid or bacterial backbone bands under the minicircle DNA digestion. (**b**) MN511C7-EF1α, MN501C7-EF1α, MC511C7-EF1α, and MC501C7-EF1α were digested by SpeI and XbaI dual restriction enzyme digestion to confirm the EF1α (1210 bp) bands. Both images were run on 0.8% agarose gels.

Based on the COL7A1 transcription and expression level from MN511C7 plasmids with different promoters, the EF1α promoter was selected for further plasmid optimization due to its higher C7 protein production levels in comparison with the other human promoter, C7P. To construct a plasmid suitable for clinical use, we cloned the C7 sequence into a MN501A1 vector which does not contain any reporter protein gene, and then replaced the CMV promoter with the EF1α promoter using the same approach carried out for MN511C7-EF1α (Figure 1a and Figure S1a). C7 and EF1α promoter sequence insertions were also confirmed by restriction enzyme digestion and sequencing analysis (Figure 4). Both MN511C7-EF1α and MN501C7-EF1α plasmids contained two attachment sites (attB and attP) that can be recognized and recombined by ϕC31 integrase. ϕC31 integrase and SceI endonuclease genes, carried by the engineered ZYCY10P3S2T *E. coli*, were switched on by addition of arabinose to the bacteria culture media. Thus, MC511C7-EF1α and MC501C7-EF1α were induced in vivo when the rest of bacteria backbone residuals were digested by SceI endonuclease (Figure S1b). After induction, we confirmed the construction of MC511C7-EF1α (12,820 bp) and MC501C7-EF1α (11,506 bp) by band size confirmation on the 0.8% agarose gels. The bands observed of linear minicircle DNA (obtained by enzymatic digestion with XbaI) were sharp and clean, without shadow of their parental plasmids (16,819 bp MN511C7-EF1α and 15,544 bp MN501C7-EF1α) or bacterial backbone (7908 bp of MN511 and 6633 bp of MN501 DNA), which demonstrated the good quality and purity of the minicircle DNA (Figure 4a).

### 2.5. In Vitro Transfection with MC501C7 in RDEBK Cells

**Figure 5:**
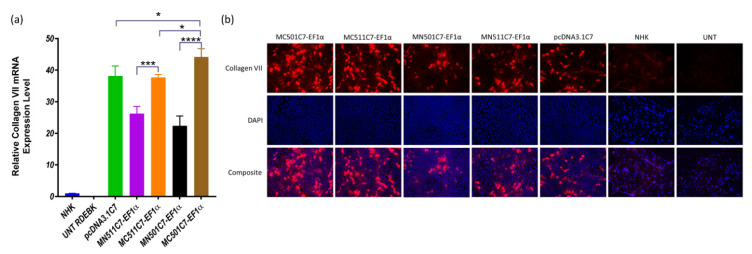
COL7A1 transcription and protein expression. (**a**) COL7A1 gene transcription level of RDEBK cells treated with MN501C7-EF1α, MC501C7-EF1α, MN511C7-EF1α. MC511C7-EF1α, and pcDNA3.1DNA plasmids were compared by RT-qPCR. Data were collected from 3 replicates of 3 independent experiments and presented as means ± SD (*n* = 3). **** *p* < 0.0001, *** *p* < 0.001, * *p* < 0.05, as compared between the different treatment conditions, normal human keratinocytes and RDEBK cells were used as positive and negative controls respectively. (**b**) Immunocytochemistry images were taken 48 h after transfection, using a 20× fluorescence microscopy objective. C7 was stained with anti-C7 antibody and AlexaFluor™ 568-labeled antibody, and the nucleus was stained with DAPI. Fluorescence microscopy images are representative of three independent experiments (*n* = 3).

The induced MC511C7-EF1α and MC501C7-EF1α plasmids were formulated with BrPERfect into nanoparticles and used to treat RDEBK cells to validate its therapeutic potential in terms of C7 production. Both minicircle systems showed much higher transcription levels of COL7A1 compared with their parental plasmids (*p* = 0.0002 and *p* ˂ 0.0001, respectively). The transcription level of MC501-EF1α was also significantly higher than that of the pcDNA3.1C7 plasmid (*p* = 0.0196). Further removal of the GFP sequence from the minicircle DNA also contributed to the enhancement of COL7A1 transcription. In addition, all treatments were statistically different from the positive control NHK cells (*p* < 0.0001), as they generated much higher C7 transcription (Figure 5a). Immunocytochemistry results also showed that minicircle DNA restored C7 expression on RDEBK cells more than their parental plasmids and pcDNA3.1C7 plasmid (Figure 5b).

## 3. Discussion

RDEB is known as a severe genetic dermatological disorder resulting from mutations in the COL7A1 gene. A mutation in this complex gene leads to a loss of function of the C7 protein, a major component of the anchoring fibrils [1]. Gene editing strategies such as CRISPR-Cas systems would be the ideal option for treating RDEB, since correcting the disease-causing mutation could offer a life-saving cure for patients, reducing the possibility of safety complications derived from the transfection vectors’ chronic application on patients’ fragile skin. However, the current in vivo low efficiency of gene editing approaches seriously hinders their clinical application [31]. Moreover, at least 774 mutations within COL7A1 have been reported in different variants of dystrophic epidermolysis bullosa [4], which makes it necessary to develop customized gene editing therapies for each patient, which is time-consuming and expensive. Therefore, a gene replacement approach which is not dependent on specific mutations still represents a promising treatment strategy for RDEB patients. 

Despite the fact that many different treatment approaches have been developed to deliver the full C7 gene, few groups have included promoter selection for viral vectors in their strategy, and research is even less common for promoter optimization in non-viral vector-mediated therapies [23,28,29,32]. In the clinic, viral promoters are the most widely used, but with the recent development of non-viral delivery systems, the full elimination of any viral sequences from the delivered DNA vector should be taken under consideration. In our previous work, a polymer-based gene replacement therapy for RDEB was developed using C7 minicircle DNA with CMV promoter to induce strong C7 expression [9,17,32,33,34], where results showed 10 to 1000-fold enhancement of transgene expression in vitro and in vivo [9,17,35,36]. The CMV promoter has been widely used most in DNA vaccines and gene therapies, as it is a strong mammalian expression promoter. However, there are some reports that gene silencing of CMV promoter-driven transgenes has been demonstrated to be associated with DNA methylation [35,36,37,38]. This transcriptional silencing of CMV promoter could occur after a certain period from 7 days to 49 days post-transduction, which varied between different cell types [22,39,40]. Therefore, to further develop our gene replacement therapy for RDEB treatment, employment of a human promoter sequence to control COL7A1 expression may help to minimize the risk of immunogenic and/or oncogenic events, and avoid any other unexpected effects from a CMV promoter.

From common mammalian promoters reported in skin therapy, we selected the human COL7A1 promoter (C7P) and the elongation factor 1α (EF1α) for tissue-specific and ubiquitous expression of the transgene, respectively [28,29,41]. In self-inactivating (SIN) retroviral vectors, both human promoters have demonstrated long-term functional correction in ex vivo gene replacement therapy [28]. The usage of C7P not only improves the security level of the construct, but also ensures that transgene expression is restricted to basal keratinocytes and dermal fibroblasts. Titeux et al. used a short sequence of 616 bp C7P rather than 1608 bp full length, due to the limitation of the viral vector capacity [28]. However, due to its good loading capacity, our non-viral gene system contains the full C7P sequence to insure that COL7A1 is completely controlled by its own promoter and full promoter’s essential response to the intracellular environment. On the other hand, EF1α is a short human housekeeping gene promoter which has strong mammalian expression. This gene expression level can even outperform the viral CMV promoter [35,42]. In order to achieve the maximum expression ability driven by the promoter, we modified the EF1α sequence according to Zheng and Baum’s study in 2014 [30]. We selected the EF1α sequence with highest expression ability and added SpeI and XbaI sites to both 3′ and 5′ sides, bringing it to a total size of 1210 bp. In our work, we compared the COL7A1 transcription and expression level from CMV, C7P and EF1α promoters in the minicircle parental plasmid first, to avoid the substantial difference between minicircle DNA purity. Although the in vivo bacterial induction system has improved the procedure of minicircle production, the induction from its parental plasmid still needs to be improved to reach good DNA quality levels in all cases. Moreover, purification of minicircle DNA is still a challenge and is a limiting step in its translation from the bench to the clinic [43].

Among the three parental plasmids with different promoters, MN511C7-EF1α showed comparable COL7A1 transcription and protein expression levels to the CMV promoter-containing plasmid after 48 h of treatment, although the CMV promoter was still slightly more efficient. The C7P promoter presented lower transcription and protein expression levels in comparison to the other two promoters. Moreover, a higher transcription level and no statistically significant difference at the C7 protein expression level was observed in MN511C7-CP7-treated RDEBK cells compared to NHK. These results may be explained by the natural intracellular control of the C7P promoter, mimicking the normal expression of C7 protein in healthy skin.

Although we finally chose the EF1α as the promoter for further work because of its outstanding gene expression level, C7P could still be a good promoter candidate for non-viral gene therapy in a setting where in vivo transfection would be efficient enough to allow production of significant levels of collagen VII, in a similar way as the normal skin. This would be the ideal situation to avoid overexpression of C7 in the already fibrotic RDEB skin, highlighting the fact that in choosing the appropriate promoter, it is important to take into account the transfection efficiency of the vector to be used for the clinical approach.

After inducing the MN511C7-EF1α parental plasmid into minicircle DNA, the transcription and expression levels for C7 were enhanced, surpassing the conventional CMV promoter-containing pcDNA3.1 DNA plasmid as well. Thus, the minicircle induction by the usage of promoters that can offer better features in terms of tissue specificity and security, but that originally do not have such high expression performances, makes it possible to obtain protein expression levels that, theoretically, can lead to the reduction of the symptoms of the disease and/or to a partial correction of it.

Due to the limited transfection achieved on fibroblasts, treatment assessments here were performed mainly focused on RDEB keratinocytes. In addition, there are several advantages of selecting keratinocytes over fibroblast cells for treatment with this non-viral skin gene therapy application, such as the fact that secretion of C7 at the dermal-epidermal junction is mostly performed by keratinocytes which contribute 90–97% of secreted C7, compared to just 3–10% from fibroblasts [39]. Furthermore, keratinocytes are more accessible than the deep dermal fibroblasts, which makes them easier to treat and to be obtained from patients. In fact, they are the target in most skin topical treatments studies [40]. However, as we mention above, there is still an urgent demand for developing a DNA delivery vector that has large payload capacity and highly efficient transfection of both dermal keratinocytes and epidermal fibroblasts. Such a vector would offer the potential to perform gene therapy in both dermis and epidermis at the same time, increasing the possibilities of finding a successful treatment for the clinic.

## 4. Materials and Methods

### 4.1. Collagen VII Minicircle Parental Plasmid Construction

The MC vector plasmids MN511A1 and MN501A1 (System Biosciences, Palo Alto, CA, USA) consist of CMV-MCS-EF1α-GFP-SV40-PolyA with the kanamycin resistance gene, and CMV-MCS-SV40-PolyA with the kanamycin resistance gene respectively. Their replication cassettes are flanked by the attP and attB sites recognized by ϕC31 integrase. The cDNA of human COL7A1 gene was cut from a plasmid named pcDNA3.1C7 which was kindly provided by Dr A. South (Thomas Jefferson University, Department of Dermatology and Cutaneous Biology, Philadelphia, PA, USA), and inserted downstream of CMV promoter in the MC vector (MN511A1/MN501A1) at the EcoRI restriction site to construct the C7 parental plasmids (MN511C7-CMV/MN501C7-CMV) (Figure 1).

The human COL7A1 promoter (C7P) sequence was obtained from ENSEMBL database (Gene: COL7A1, ENSG00000114270), and human EF1α promoter sequence was obtained from the plasmid pEAK8 (Edge Biosystems, San Jose, CA, USA). Spel and XbaI restriction enzyme sites were added to both promoters at the 5′ and 3′ ends, respectively. Then, the promoter sequence was synthesized by Eurofins Scientific (Luxembourg, Luxembourg) in pEX-K plasmids containing kanamycin resistance gene, as pEX-K-C7P and pEX-K-EF1α. Parental plasmids (PPC7) using human promoters were constructed by replacing the CMV promoter of MN511C7-CMV/MN501C7-CMV with the above human promoters between the Spel and XbaI restriction enzyme sites (Figure 1).

### 4.2. Parental Plasmid/Minicircle Production and Purification

The PPC7 was transformed into competent ZYCY10P3S2T *Escherichia coli* cells (System Biosciences) by heat-shock. A single bacterial colony was inoculated in 5 mL of Luria-Bertani broth (LB) (Sigma Aldrich, St. Louis, MO, USA) with kanamycin (500 µg/mL) at 30 °C with shaking at 250× *g* rpm for 5 h and considered as the starting culture. Afterwards, 100 μL of the starting culture was added to 400 mL terrific broth (TB, Sigma-Aldrich) with kanamycin (500 µg/mL) at 30 °C with shaking at 250× *g* rpm for around 15 h. Then, bacteria were centrifuged at 4700× *g* rpm for parental plasmid extraction or progress to minicircle induction.

The collagen VII minicircle DNA (MCC7) was produced following a modified manufacturer’s instruction. The minicircle DNA induction mix, consisting of 400 mL LB, 16 mL 1 N NaOH and 420 μL 20% L-arabinose (System Biosciences), was added into the TB bacterial culture [41]. Then, bacteria were incubated at 250× *g* rpm for an additional 5 h (at 30 °C for 4 h, 37 °C for 1 h). Bacteria which should contain MC were centrifuged at 4700× *g* rpm and progressed to MC extraction. To check the MC quality, it was extracted from 5 mL of bacteria culture using the QIAprep Spin Miniprep kit (QIAGEN, Germantown, MD, USA) following the user manual. Then, 0.5 to 1 µg of MC DNA was digested by XbaI restriction enzyme and then run on 0.8% agarose gel. After the confirmation of good quality, the MC was further extracted from the rest of the bacterial culture using QIAGEN Plasmid Mega kit (QIAGEN).

### 4.3. Cell Culture

Immortalized primary human RDEB keratinocytes (RDEBK) and fibroblasts (RDEBF) were kindly provided by Dr. F. Larcher (Centro de Investigaciones Energéticas, Medioambientales y Tecnológicas-CIEMAT, Madrid, Spain). Immortalized RDEBF cells were cultured in Dulbecco’s Modified Eagle Medium (DMEM) 6429 (Sigma-Aldrich) with 10% fetal bovine serum (FBS), (Thermo Fisher Scientific, Waltham, MA, USA) and 1% penicillin/streptomycin (Thermo Fisher Scientific). Normal human keratinocytes (NHK) (Promocell, Heidelberg, Germany) and immortalized RDEBK cells were cultured using standard cell culture techniques in keratinocyte growth complete FAD medium (KCa) as described by Bonafont et al. [44] and incubated at 37 °C and 5% CO_2_ in a humidified incubator. Cells were seeded at cell density of 31,250 cells/cm^2^ in 96-well plates for GFP expression imaging and flow cytometry, in 48-well plates for ICC and in 12-well plates for WB and qPCR.

### 4.4. Cell Transfection

Cells were seeded 24 h prior to transfection when cells reached 70–80% confluence, transfections were carried out with three commercial transfection reagents: BrPERfect DNA transfection reagent (Branca Bunús Ltd., Dublin, Ireland), Lipofectamine™ 3000 (Thermo Fisher Scientific), and jetPEI (Polyplus Transfection, Illkirch-Graffenstaden, Strasbourg, France). BrPERfect DNA transfection reagent is a degradable polymer derivative, designed by our group and commercially available. DNA and BrPERfect were diluted in 25 mM sodium acetate at DNA to BrPERfect weight/weight (*w*/*w*) ratio of 30 to 1, then mixed at a 1:1 volume/volume (*v*/*v*) ratio and incubated at room temperature for 10 min before adding it to the corresponding cell culture media. Both RDEBK and RDEBF cells were transfected with plasmids at 5 ng/µL formulated with BrPERfect. DNA-lipid complex was carried out with Lipofectamine™ 3000 (plasmids at 2 ng/µL), and DNA-polymer complex transfection was done using JetPEI (plasmids at 1.67 ng/µL) following the manual instructions. Expression of the reporter gene green fluorescent protein (GFP) was visualized 48 h post-transfection with gWiz-GFP commercial plasmid (Aldevron, Fargo, ND, USA) or MN511C7 plasmid using an Olympus IX81 fluorescence microscope (Olympus, Tokyo, Japan). The intensity of GFP fluorescence was analysed and semi-quantified using the ImageJ software (NIH, Bethesda, MD, USA).

### 4.5. Cell Viability

Cell viability was assessed 48 h post-transfection. Medium was removed and cells were washed with 100 μL of Hank’s Balanced Salt Solution (HBSS, Sigma Aldrich) per well. Then, 100 μL of alamarBlue™ (Thermo Fisher Scientific) working solution (10% alamarBlue™ in HBSS) were added to each well and incubated for 2 h under normal cell culture conditions previously described. Absorbance was recorded at 570 nm and 600 nm by a SpectraMax M3 multi-plate reader (Molecular Devices, San Jose, CA, USA). Untreated cells were used to normalize fluorescence values and set as 100% viable. Wells containing only alamarBlue™ reagent were subtracted as background prior to obtaining the % of cell viability, calculated as follows:

(Absorbance of treated cells/Absorbance of untreated) × 100%.

### 4.6. RT-qPCR

RT-qPCR was performed to quantify the COL7A1 mRNA (exon 64) expression. Cells were transfected by BrPERfect as mentioned above and the total RNA of transfected and untreated cells was extracted using RNeasy Mini Kit (QIAGEN) 48 h post-transfection. Then, 0.1 μg of total RNA from each group were used to synthesize the first-strand cDNA with the primer Oligo (dT)20 (50 μM) according to the protocol of SuperScript III First-Strand Synthesis SuperMix. Afterwards, 1 μL of the final complementary DNA (cDNA) product was added to 9 μL of reaction mix (0.5 μL C7 exon64 TaqMan primer (Hs00164310_m1) (Thermo Fisher Scientific), 0.5 μL Human Cyclophilin A (PPIA) Endogenous Control TaqMan primer (4326316E) (Thermo Fisher Scientific), 5 μL TaqMan PCR mix (4369016) (Thermo Fisher Scientific), and 3 μL RNase-free water), which were loaded on a 384-well plate. Each sample was measured in triplicate. For COL7A1 quantitative gene expression, human PPIA was used as the endogenous control. Comparative CT values, TaqMan reagents and QuantStudio 7 Flex System were set up for the experiments. Results were analysed with the QuantStudio Real-Time PCR Software.

### 4.7. Western Blotting of Collagen VII

After 48 h post-transfection, cell lysates were harvested using radioimmunoprecipitation assay (RIPA) buffer containing protease cocktail inhibitor (cOmplete™ Mini Protease Inhibitor Cocktail, Roche, Basel, Switzerland). Lysates were frozen at −20 °C overnight and then centrifuged for 10 min at 14,800× *g* rpm at 4 °C. Then, protein supernatants were moved to fresh 1.5 mL tubes. Protein samples were quantified using Bicinchoninic Acid (BCA) Protein Assay (Thermo Fisher Scientific) and separated in 3–8%-gradient Tris-acetate gels (Thermo Fisher Scientific) to be later transferred to a nitrocellulose membrane at 90 V for 90 min. For detection of C7, a rabbit anti-C7 antibody (kindly provided by Dr. Alexander Nyström) (University of Freiburg, Freiburg, Germany) was used at a dilution of 1:5000 [45]. As loading control, human GAPDH antibody (ab8245) (Abcam, Cambridge, UK) was used at a dilution of 1:5000 as well. The secondary antibodies for the corresponding primary antibodies, anti-rabbit IgG7074 (Cell Signaling Technology, Danvers, MA, USA) and anti-mouse IgG-7076 (Cell Signaling Technology), were used at 1:2000 dilution and 1:5000 dilution. Protein bands were developed using the Pierce™ ECL Western Blotting Substrate (Thermo Fisher Scientific) and the iBright CL750 Imaging System (Thermo Fisher Scientific). Semi-quantitative analysis of C7 bands was performed using ImageJ Fiji software (NIH). All C7 bands were normalized to the loading control GAPDH.

### 4.8. Flow Cytometric Analysis

After 48 h post-transfection, cells were detached from the plate, and fixed in Fixation Buffer (BioLegend, San Diego, CA, USA) at room temperature for 20 min in the dark. Afterwards, cells were permeabilized by resuspension in Intracellular Staining Permeabilization Wash Buffer (BioLegend) and blocked in Stain Buffer (FBS) (BD Biosciences, San Jose, CA, USA) for 15 min. The primary anti-C7 antibody (kindly provided by Dr. Alexander Nyström) was added at a dilution of 1:1000 to be later incubated with the AlexaFluor™ 568-labeled secondary antibody (A11036) (Thermo Fisher Scientific) at a dilution of 1:200. Cells were analysed using CytoFLEX Flow Cytometer (Beckman Coulter Life Sciences, Indianapolis, IN, USA). Data analysis was performed using the Cytexpert software.

### 4.9. Immunocytochemistry

Cells were pre-seeded in 48-well plates containing 9 mm coverslips. These coverslips were washed three times in ice-cold phosphate buffer saline (PBS, Thermo Fisher) and then fixed with ice-cold acetone:methanol for 20 min at −20 °C. Samples underwent three washes with PBS, to be then blocked in 3% bovine serum albumin (BSA, Sigma Aldrich) and incubated with a rabbit anti-C7 primary antibody (kindly provided by Dr. Alexander Nyström) at a dilution of 1:5000. The coverslips were then incubated with AlexaFluor™ 568-labeled secondary antibody (A-11031) (Thermo Fisher Scientific) at 1:800 dilution, mounted on microscope slides with Fluoroshield (Abcam) mounting medium with DAPI, and imaged using an Olympus IX83 microscope (Olympus).

### 4.10. Statistical Analysis

All data are represented with the mean ± standard deviation (±SD), normally performing a minimum of three independent experiments. Analysis was carried out by one-way analysis of variance (ANOVA) with Dunnett’s multiple comparison tests for single variable, and two-way analysis of variance (ANOVA) with the Tukey’s multiple comparison test for grouped variables, through the software GraphPad Prism 8.0 (San Diego, CA, USA). *p* values of less than 0.05 were considered significant (* *p* < 0.05, ** *p* < 0.01, *** *p* < 0.001, **** *p* < 0.0001). Statistical significance was reported in the figure legends.

## 5. Conclusions

In this study, we designed and generated two new minicircle plasmids encoding full COL7A1 gene regulated by different human promoters and tested their potential to be used in gene replacement therapy for RDEB. Although changing a strong viral promoter into a human promoter reduced the gene expression initially, the minicircle system induction in the case of the EF1α-containing plasmid led to higher protein expression levels in comparison with the normal pcDNA3.1 vector using CMV promoter, with improved safety profile as well. Moreover, and based on results with MN511C7-C7P, usage of tissue-specific promoters such as C7P is also a promising alternative in case of achieving high transfection levels in vivo, as production of C7 is similar to normal keratinocytes. Therefore, our newly developed non-viral minicircle DNA gene replacement strategy has demonstrated promise for application in RDEB treatment and could also be applied to other genetic diseases.

## Data Availability

All data generated or analyzed during this study are included in this published article and its supplementary information file.

## Supplementary Materials

The following are available online at https://www.mdpi.com/article/10.3390/ijms222312774/s1.
