# Supplementary material for: Development of Minicircle Vectors Encoding COL7A1 Gene with Human Promoters for Non-Viral Gene Therapy for Recessive Dystrophic Epidermolysis Bullosa
Source: Int J Mol Sci. 2021 Nov 26;22(23):12774. doi: 10.3390/ijms222312774 (PMC8657908; doi:10.3390/ijms222312774)
Supplement: Supplementary file 1 [file ijms-22-12774-s001.zip › ijms-1443528-supplementary.pdf]

Table S1: Plasmids used in this study.

Plasmid	Size (bp)	Collagen VII	Promoter of Collagen VII	GFP	Promoter of GFP	Bacterial Backbone
MN511A1	7063	N	-	Y	EF1α	Y
MN501A1	5788	N	-	N	-	Y
MN511C7-CMV	15974	Y	CMV	Y	EF1α	Y
MN501C7-CMV	14699	Y	CMV	N	-	Y
MN511C7-TSP	15414	Y	TSP	Y	EF1α	Y
MN511C7-EF1α	16819	Y	EF1α	Y	EF1α	Y
MN501C7-EF1α	15544	Y	EF1α	N	-	Y
MC511C7-EF1α	12820	Y	EF1α	Y	EF1α	N
MC501C7-EF1α	11506	Y	EF1α	N	-	N
pcDNA3.1C7	14269	Y	CMV	N	-	Y
gWiz-GFP	5757	N	-	Y	CMV	Y

Y: with the protein coding gene; N: without the protein coding gene; -: no answer

Table S2: Primers used for sequencing in this study.

Primer	Sequence (From 5′ to 3′)	GC Content (%)	Tm (°C)
C7-F	ACGCTGTTTTGACCTCCATAGAAG	45.8	61
C7-R	TGTGCTCCGCGTCTCTCATG	60	61.4
C7P-F	TAACTCCATCTAAGGTCACA	40	53.2
C7P-R	CAGGTCACTCTCTCCCTGTGCT	59.1	64
EF1α-F	ATGGTCGAGACTCAGCGGC	63.2	61
EF1α-R	CTGCTCCAGAGAAAGGCAGCA	57.1	61.8

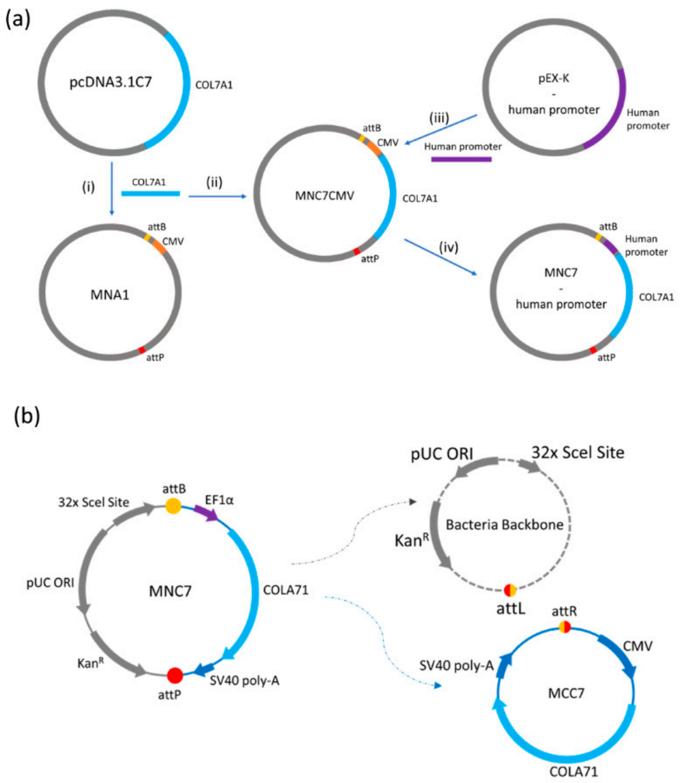

Figure S1: Scheme of COL7A1 plasmid construction and minicircle induction. (**a**) Workflow of minicircle parental plasmid construction with three different promoters. (i) Excise the full COL7A1 sequence from pcDNA3.1C7 plasmid by EcoRI restriction enzyme. (ii) Insertion of the obtained COL7A1 sequence into parental plasmid vectors (MN511A1 or MN501A1) to generate MN511C7-CMV or MN501C7-CMV respectively. (iii) Excise the human promoters from pEX-K plasmid at the pre-added SpeI and XbaI restriction enzyme sites. (iv) Removal of the CMV promoters in MN511C7-CMV or MN501C7-CMV and ligation of the human promoters to produce MN511C7-C7P, MN511C7-EF1α and MN501C7-EF1α. (**b**) Scheme of minicircle induction. Recombination of the parental plasmid by ϕC31 integrase at attB and attP sites and production of a minicircle DNA with COL7A1 sequence and independent bacterial backbone residual DNA. Both parental plasmid and bacterial backbone were digested by SceI endonuclease during the bacterial incubation and only minicircle DNA with the gene of interest was extracted.

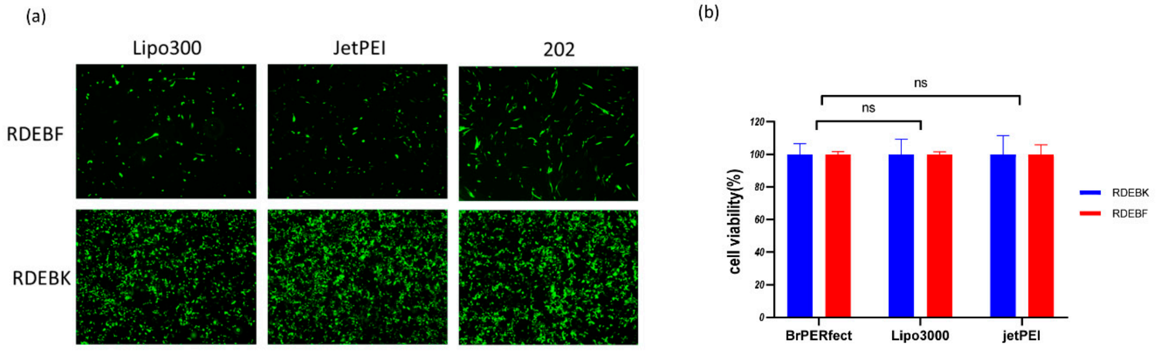

Figure S2: DNA transfection reagent selection in RDEBF cells and comparison with RDEBK cells. (**a**) Fluorescence microscopy images of GFP expression from transfected RDEBF and RDEBK cells with gWiz-GFP plasmid by using BrPERfect, Lipofectamine 3000 and jetPEI transfection reagents at 48 hrs post-transfection, using a 4X objective. (**b**) Cell viability of transfected RDEBF and RDEBK cells with gWiz-GFP was tested using alamarBlue™. Data were collected from 3 replicates of 3 independent experiments and presented as means ± SD (n = 3). ns: not significant, as compared to BrPERfect.

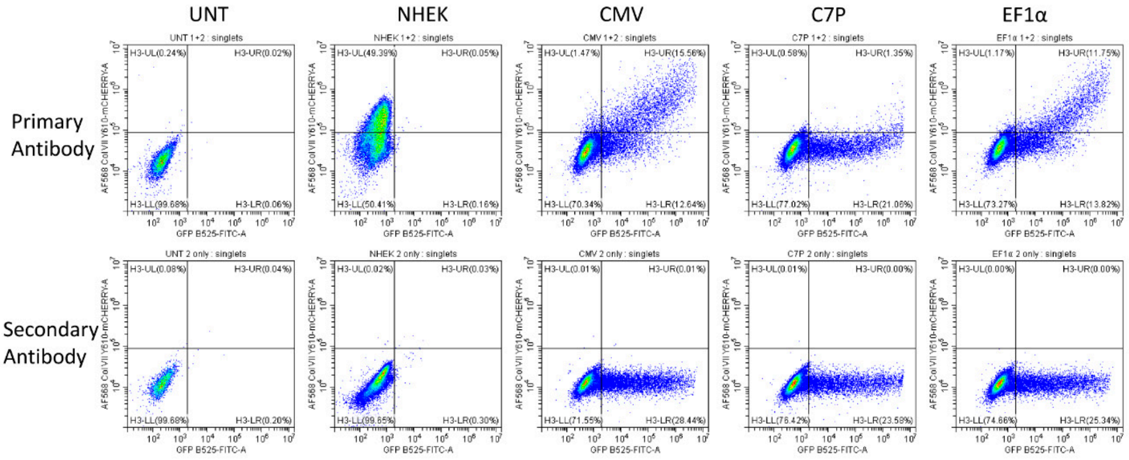

Figure S3: GFP and C7 positive cells gating strategy for flow cytometry. Both C7 and GFP expression in RDEBK cells transfected with the three MN511C7 plasmids containing different promoters were evaluated. NHK cells were used as C7 positive control and untreated RDEBK cells used as negative control for C7 and GFP. RDEBK cells transfected with pcDNA3.1 plasmid were used as C7 positive but GFP negative control. UNT: untreated RDEBK cells; NHK: normal human epidermal keratinocytes; CMV: MN511C7-CMV plasmid; C7P: MN511C7-C7P plasmid; EF1α: MN511C7-EF1α plasmid, pc3.1: pcDNA3.1C7 plasmid.
